# Nested PCR Approach for *petB* Gene Metabarcoding of Marine *Synechococcus* Populations

**DOI:** 10.1128/spectrum.04086-22

**Published:** 2023-03-06

**Authors:** Denise Rui Ying Ong, Andrés Gutiérrez-Rodríguez, Laurence Garczarek, Dominique Marie, Adriana Lopes dos Santos

**Affiliations:** a Asian School of the Environment, Nanyang Technological University, Singapore; b National Institute of Water and Atmospheric Research (NIWA), Wellington, New Zealand; c Sorbonne Université, CNRS, UMR 7144 (AD2M), Station Biologique de Roscoff, Roscoff, France; Connecticut Agricultural Experiment Station

**Keywords:** marine picocyanobacteria, marine *Synechococcus*, *petB*, nested PCR, metabarcoding

## Abstract

The molecular diversity of marine picocyanobacterial populations, an important component of phytoplankton communities, is better characterized using high-resolution marker genes than the 16S rRNA gene as they have greater sequence divergence to differentiate between closely related picocyanobacteria groups. Although specific ribosomal primers have been developed, another general disadvantage of bacterial ribosome-based diversity analyses is the variable number of rRNA gene copies. To overcome these issues, the single-copy *petB* gene, encoding the cytochrome *b*_6_ subunit of the cytochrome *b*_6_*f* complex, has been used as a high-resolution marker gene to characterize *Synechococcus* diversity. We have designed new primers targeting the *petB* gene and proposed a nested PCR method (termed Ong_2022) for metabarcoding of marine *Synechococcus* populations obtained by flow cytometry cell sorting. We evaluated the specificity and sensitivity of Ong_2022 against the standard amplification protocol (termed Mazard_2012) using filtered seawater samples. The Ong_2022 approach was also tested on flow cytometry-sorted *Synechococcus* populations. Samples (filtered and sorted) were obtained in the Southwest Pacific Ocean, from subtropical (ST) and subantarctic (SA) water masses. The two PCR approaches using filtered samples recovered the same dominant subclades, Ia, Ib, IVa, and IVb, with small differences in relative abundance across the distinct samples. For example, subclade IVa was dominant in ST samples with the Mazard_2012 approach, while the same samples processed with Ong_2022 showed similar contributions of subclades IVa and Ib to the total community. The Ong_2022 approach generally captured a higher genetic diversity of *Synechococcus* subcluster 5.1 than the Mazard_2012 approach while having a lower proportion of incorrectly assigned amplicon sequence variants (ASVs). All flow cytometry-sorted *Synechococcus* samples could be amplified only by our nested approach. The taxonomic diversity obtained with our primers on both sample types was in agreement with the clade distribution observed by previous studies that applied other marker genes or PCR-free metagenomic approaches under similar environmental conditions.

**IMPORTANCE** The *petB* gene has been proposed as a high-resolution marker gene to access the diversity of marine *Synechococcus* populations. A systematic metabarcoding approach based on the *petB* gene would improve the characterization/assessment of the *Synechococcus* community structure in marine planktonic ecosystems. We have designed and tested specific primers to be applied in a nested PCR protocol (Ong_2022) for metabarcoding the *petB* gene. The Ong_2022 protocol can be applied to samples with low DNA content, such as those obtained by flow cytometry cell sorting, allowing the simultaneous assessment of the genetic diversity of *Synechococcus* populations and cellular properties and activities (e.g., nutrient cell ratios or carbon uptake rates). Our approach will allow future studies using flow cytometry to investigate the link between ecological traits and taxonomic diversity of marine *Synechococcus*.

## INTRODUCTION

Marine photosynthetic bacterioplankton is dominated by two genera of picocyanobacteria: *Prochlorococcus* and *Synechococcus*. *Synechococcus* is a polyphyletic genus comprising both marine and freshwater lineages (1). They contribute up to 16% of the net marine primary productivity (2, 3). Changes in the physicochemical properties of marine waters induced by climate change are predicted to have an impact on the distribution of phytoplankton groups, including *Synechococcus* (4). Given the major role of these phytoplankton groups in marine carbon cycling and the ocean food web (5, 6), an extensive assessment of *Synechococcus* community genetic diversity at spatial and temporal scales as well as the ecological traits of this community is critical to understand the future evolution of this group under the ongoing global change.

While studies using the 16S rRNA gene have revealed some degree of diversity within *Prochlorococcus* and *Synechococcus* populations (7–9), the relatively low sequence divergence between closely related clades or species and the variability in copy number of rRNA genes (8, 10) have prompted the scientific community to use high-resolution marker genes to analyze the genetic diversity within these groups of picocyanobacteria (e.g., *ntcA* [11], *petB* [12], internal transcribed spacer [ITS] [13], and *rpoC1* [14]). Although most of these gene markers have been amplified from natural *Synechococcus* populations by standard PCR amplification, others like *rpoC1* require a nested PCR approach (15, 16).

Nested and semi-nested PCR approaches involve two sequential amplification reactions with different pairs of primers. These amplification methods are often applied to increase the sensitivity and/or specificity of the reaction. They are also particularly useful for samples with low nucleic acid concentrations (15, 17). For example, metabarcoding of flow cytometry (FCM)-sorted phytoplankton populations typically require a nested or semi-nested PCR amplification approach because sorted cells are in general in very low abundance compared to those collected in filtered samples (18, 19).

Metabarcoding is a suitable technique to assess the taxonomic diversity of flow cytometry-sorted populations with high sensitivity (e.g., trace concentrations of DNA can be PCR amplified and sequenced) and for many samples, including *Synechococcus*. Although alternative approaches of metagenome or whole-genome sequencing could similarly obtain the taxonomic diversity without metabarcoding limitations of primer and amplification biases, these approaches require a much larger sequencing depth and sample processing time, which would increase the cost and time needed (20). Whole-genome sequencing would instead be more advantageous when applied to functional diversity and population genetics (21, 22). Flow cytometry-sorted *Synechococcus* cells have provided important information about their metabolism. These sorted populations have been used to measure cellular properties, such as nutrient stoichiometric and isotopic compositions (23, 24), and cellular activities, such as glucose (25), phosphorus (26), nitrogen (27, 28) and CO_2_ uptake rates (5, 25, 27, 29–31). However, only a few of these studies combined quantitative cell measurements with a fine taxonomic identification of the sorted populations by molecular methods (clone library sequencing and fluorescence *in situ* hybridization only) (29–31).

The *petB* gene, encoding the cytochrome *b*_6_ subunit of the cytochrome *b*_6_*f* complex, possesses several features that make this locus a good candidate for a gene marker. *petB* is a single-copy gene, therefore reducing amplification bias (12). It is highly conserved in length and sequence, allowing the obtained reads to be easily aligned (12), and has a comprehensive reference nucleotide sequence database that encompasses most of the genetic diversity identified within marine *Prochlorococcus* and *Synechococcus* populations (32). Few specific primers targeting *petB* gene amplification are currently available in the literature. Mazard et al. (12) developed the original primer set, petB-F and petB-R. Ohnemus et al. (33) modified the original pair by increasing their degeneracy (with degeneracy of up to 32 for both forward and reverse primers) in order to expand the diversity coverage of marine picocyanobacterial groups. The annealing regions of these primer sets are very similar and the degeneracy too high, precluding their use in nested or semi-nested PCR amplification approaches.

With the objective of establishing a new nested PCR methodology to survey the diversity of flow cytometry-sorted *Synechococcus* populations, we have designed and tested a new pair of primers (petB-50F and petB-634R) targeting the *petB* gene locus. Our approach to assess the diversity of marine *Synechococcus* populations by metabarcoding *petB* includes a nested PCR amplification method (termed Ong_2022) which combines the newly developed pair (petB-50F and petB-634R) with the original primer set (petB-F and petB-R) established by Mazard et al. (12). We have evaluated the sensitivity and specificity of our nested PCR protocol against those of the standard PCR protocol with petB-F and petB-R (termed Mazard_2012) on *Synechococcus* populations recovered from filtered seawater samples and populations sorted by flow cytometry from the same water parcels as the filtered samples.

## RESULTS

### Primer design and PCR amplification.

The design of the new primers followed three main criteria. First, the primers must contain none or a very low number of mismatches (<3) across all *Synechococcus* reference sequences present in the database in order to avoid bias in the amplification. Second, the resulting amplicon sequence must contain enough nucleotide variation to differentiate between the clades and subclades and therefore provide good taxonomic resolution. Last, the amplicon length ideally should be less than 550 bp, so sequencing could be performed with Illumina 2 × 300 bp chemistry.

While the first two benchmarks were fulfilled with the new primer set, petB-50F and petB-634R, the final amplicon length criterion could not be achieved. In the Ong_2022 protocol, the new primers combined with petB-F and petB-R from Mazard et al. (12) (petB-634R during the first round and petB-50F during the second round of amplification) produced an amplicon with a length of 571 bp (from position 50 to 618 of the *petB* gene of the *Synechococcus* sp. strain WH8109 reference; accession number CP006882) while the standard Mazard_2012 PCR generates a fragment of 597 bp (Table 1). There was no overlap between the forward and reverse reads obtained with both standard and nested PCRs. To solve this problem, during sequence processing, we merged our reads by adding 10 degenerate base ‘N’ (for any one base) at their 3′ end. Amplicons without overlap have been successfully processed with the DADA2 pipeline using this bioinformatic maneuver, which does not significantly influence k-mer-based taxonomic classification such as the naive Bayesian classifier implemented in DADA2 (34, 35). Moreover, concatenated sequences (i.e., joined without overlap) for longer amplicons were shown to improve taxonomic recovery and classification over merged sequences (36).

**TABLE 1:** Primers sequence and PCR conditions used for amplification^*a*^

Method	PCR type	Round	Amplicon length (bp)	Primer name	Direction	Sequence	Gene positions	GC%	Deg	*T* (°C)	Reference
Mazard_2012	Standard	1	597	petB-F	Forward	TACGACTGGTTCCAGGAACG	22–41	55	0	55	Mazard et al. (12)
				petB-R	Reverse	GAAGTGCATGAGCATGAA	601–618	44	0		Mazard et al. (12)
Ong_2022	Nested	1	613	petB-F	Forward	TACGACTGGTTCCAGGAACG	22–41	55	0	59	Mazard et al. (12)
				petB-634R	Reverse	GCTTVCGRATCATCARGA	615–634	47	12		This study
		2	569	petB-50F	Forward	CAGGACATYGCTGAY	50–66	50	8	55	This study
				petB-R	Reverse	GAAGTGCATGAGCATGAA	601–618	44	0		Mazard et al. (12)

a Nested PCR approach for *petB* gene metabarcoding of marine *Synechococcus* populations. Based on *Synechococcus* sp. strain WH 8109 (CP006882), the *petB* gene is located between positions 440919 and 441575 of the genome. The total length of the *petB* gene is 657 bp. The amplicon length includes the primers. The position of the primer in *petB* gene is indicated in reference to the first nucleotide of the gene. Deg, degeneracy; *T* (°C), annealing temperature.

A total of 71 DNA samples extracted from filtered seawater and 18 flow cytometry-sorted samples were obtained from subtropical (ST1 and ST2) and subantarctic (SA1, SA2, and SA3) waters along the Chatham Rise (see Fig. S1 and Table S1 in the supplemental material). Among the DNA-extracted samples, an amplification product was obtained for all environmental samples using Ong_2022 nested PCR, and one sample could not be amplified by the standard Mazard_2012 method. Hence, from this point, the comparison between Mazard_2012 and Ong_2022 approaches included only the 70 samples successfully amplified by both methods (Table S1). The results from sorted samples were obtained only with the Ong_2022 PCR since none of the sorted samples were amplified with success by standard PCR.

Following sequence processing, the total numbers of reads retained in the final data set were about 49% and 23% of the initial number of reads generated with Mazard_2012 and Ong_2022, respectively (Table 2). The chimera detection step removed 66% of reads obtained with Ong_2022 and 35% obtained with Mazard_2012 after forward and reverse reads were merged (Table 2). The resulting mean number of reads obtained with the standard PCR was almost three times higher than the mean number of reads obtained with the nested PCR. However, the number of amplicon sequence variants (ASVs) generated with the nested PCR was higher (6,307) than that generated with the Mazard_2012 standard reaction (4,716) (Table 2).

**TABLE 2:** Number of reads and ASVs per sample after each sequence processing step using DADA2 on filtered samples obtained by the Mazard_2012 and Ong_2022 approaches

Parameter	DADA2 step	Value (mean ± SD) for:	
		Mazard_2012	Ong_2022
No. of reads/sample	1. Initial	57,321 ± 5,918	46,382 ± 4,614
	2. Remove primers	47,299 ± 5,718	34,524 ± 4,174
	3. Filter and trim	45,072 ± 6,006	32,274 ± 4,327
	4. Merge	43,653 ± 6,015	30,903 ± 4,402
	5. Remove chimera	28,237 ± 5,908	10,500 ± 2,843
No. of ASVs			
Total		4,716	6,307
Per sample		361 ± 64	217 ± 63

### Community composition from filtered samples.

**FIG 1:**
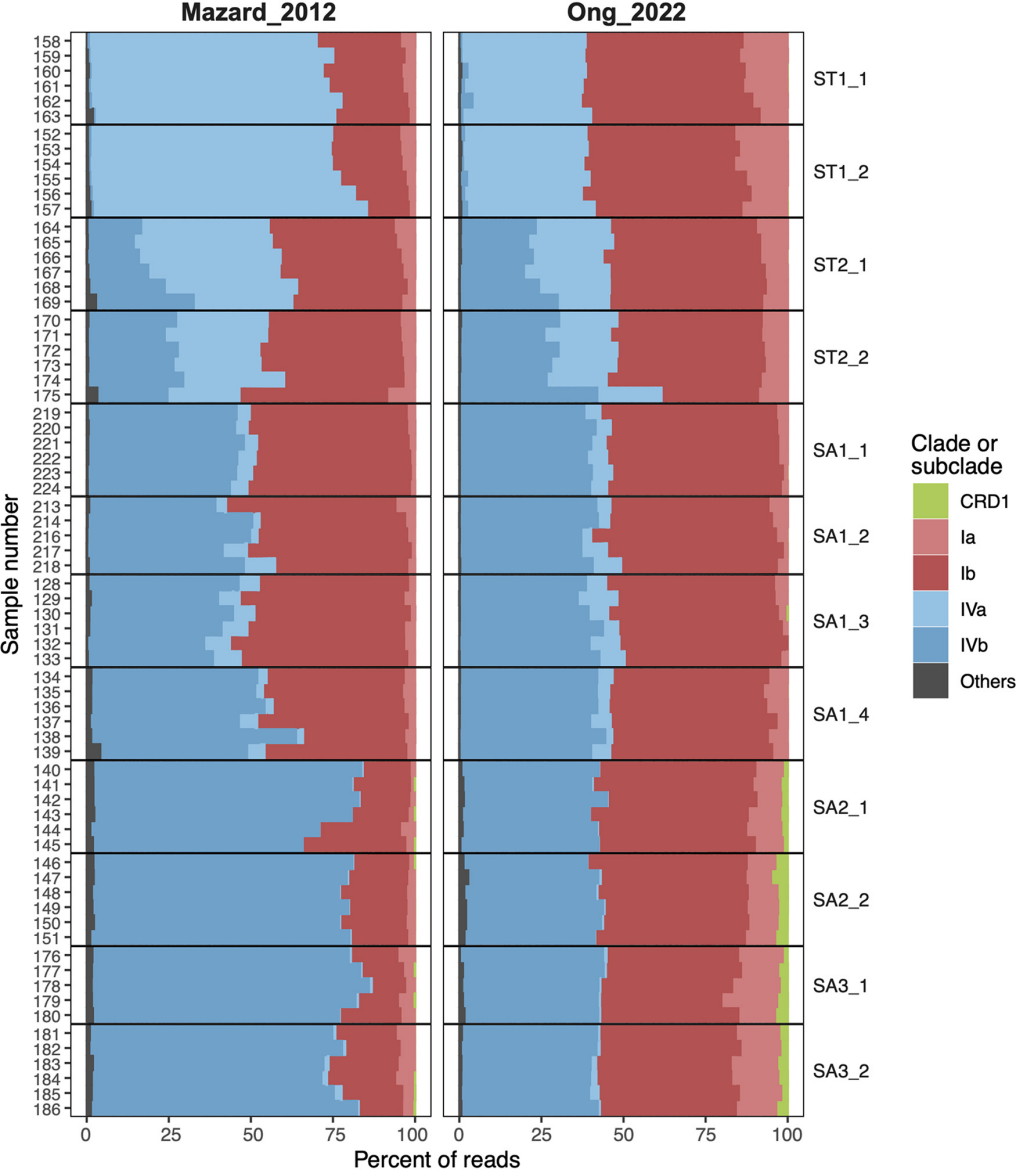
*Synechococcus* taxonomic composition at clade and subclade level from filtered samples obtained with the Mazard_2012 and Ong_2022 approaches. Samples were grouped by cycles and ordered across a spatial gradient, from subtropical (ST) to subantarctic (SA) cycles. Samples from all depths were combined.

**FIG 2:**
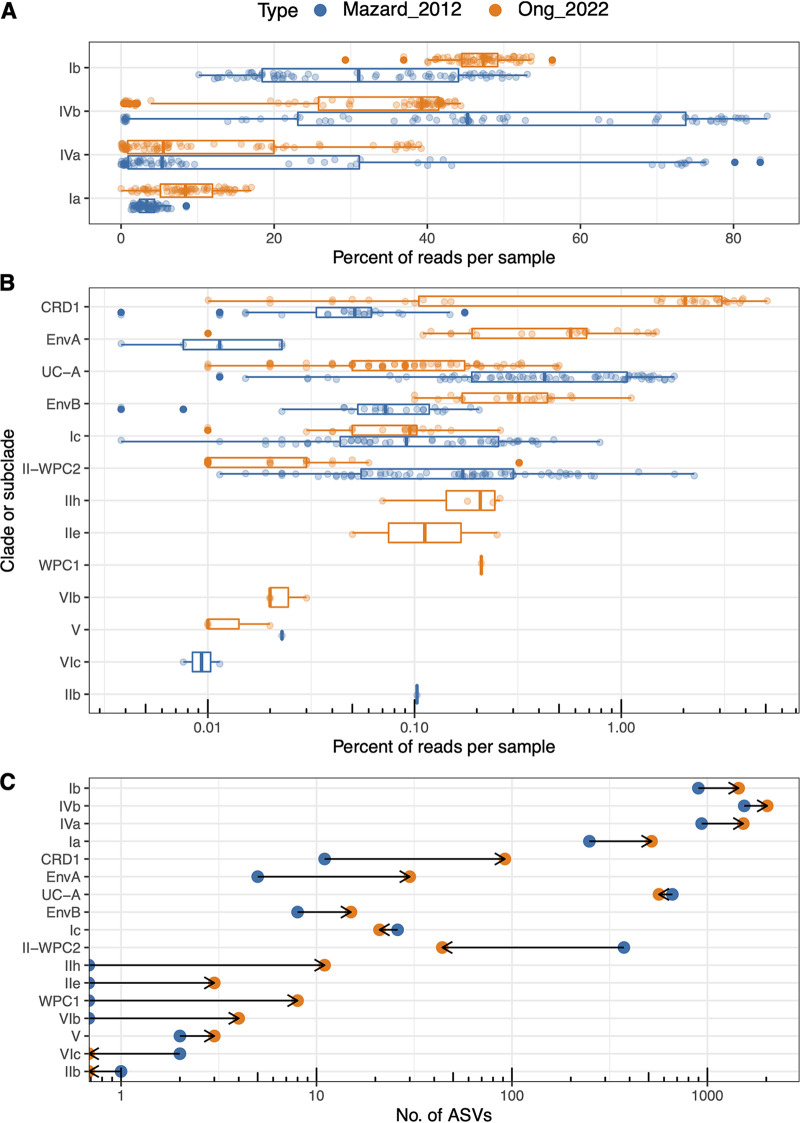
Percentage of *Synechococcus* reads in each filtered sample for major subclades (A) and minor clades and subclades (B) and total number of ASVs (C) for each clade and subclade obtained by the Mazard_2012 and Ong_2022 methods. Clades and subclades are arranged in descending order according to the mean percentage of reads with the Ong_2022 method.

Four major *Synechococcus* subclades that were present in almost all samples were Ia, Ib, IVa, and IVb (Fig. 1). While these subclades represented nearly 99% of the total number of reads obtained by both approaches, the percentage of reads per sample for each of the major subclades differed between the amplification methods: Mazard_2012 produced a higher proportion of reads from subclades IVa and IVb, while Ong_2022 amplified a higher proportion of subclade Ia and Ib sequences (Fig. 2A). Yet, patterns of community composition, such as the higher relative abundance of clade IVa in ST than in SA waters, were captured by both approaches. Among the seven minor clades and subclades present in both amplifications (CRD1, EnvA, EnvB, UC-A, Ic, II-WPC2, and V), the proportions of reads of clades CRD1, EnvA, EnvB, and V were higher in Ong_2022 than in Mazard_2012 (Fig. 2B; Table S2). Six clades and subclades with low contributions to the total community were amplified only by either the Ong_2022 (WPC1, IIh, IIe, and VIb) or the Mazard_2012 (IIb and VIc) PCR method (Fig. 2B).

A paired sample Wilcoxon test indicated that the median percentage of reads for each clade and subclade in Mazard_2012 was significantly different from that in Ong_2022, except for clade V, which was present at a very low proportion (Table S2). The mean percentages of reads for subclades Ia and Ib with Ong_2022 were higher than those with Mazard_2012 (8.51% and 46.73% versus 3.47% and 31.04%, respectively). The mean percentages of reads for subclades IVa and IVb in Ong_2022 were lower than those in Mazard_2012 (11.96% and 31.40% versus 20.39% and 44.04%, respectively) (Table S2). Among clades and subclades with minor contributions of reads to the total community, the mean percentages of reads of clade UC-A and subclades Ic and II-WPC2 were higher in Mazard_2012 than in Ong_2022 (Fig. 2B; Table S2). Clade UC-A was detected in all samples across both amplification types, but the average percentage of reads was higher in Mazard_2012 (0.63%) than in Ong_2022 (0.13%). Subclades II-WPC2 and Ic were present in higher proportions and detected in more samples with Mazard_2012 than with Ong_2022. For subclade II-WPC2, the average percentage of reads in Mazard_2012 was 0.26% and found in 67 samples, compared to Ong_2022, with an average percentage of reads of 0.01% and found in 27 samples. The average percentage of reads of subclade Ic in Mazard_2012 was 0.12% and found in 51 samples, compared to Ong_2022, with an average of 0.03% and found in 20 samples. The average percentages of reads of clades CRD1, EnvA, EnvB, and V were higher in Ong_2022 (0.93%, 0.18%, 0.11%, and 0.0006%, respectively) than in Mazard_2012 (0.02%, 0.001%, 0.03%, and 0.0003%, respectively) (Fig. 2B; Table S2). Clades CRD1, EnvA, and V were present in more samples in Ong_2022 (35, 21, and 3 samples, respectively) than in Mazard_2012 (24, 5, and 1 samples, respectively). Clade EnvB was detected in the same 23 samples for both amplification types. Clade WPC1 and subclades IIh, IIe, and VIb were present only in Ong_2022, although they were found in low proportions (less than 0.21% of reads in a sample) and only in 1 to 4 samples each. Subclades VIc and IIb were present only in Mazard_2012 but in very low proportions (less than 0.10% of reads in a sample) and only in 1 to 2 samples (Table S2).

Most clades and subclades had a higher number of ASVs in Ong_2022 than in Mazard_2012 (Fig. 2C). Subclades with a higher mean percentage of reads also had a higher number of ASVs. The exceptions were clade UC-A and subclades Ic and II-WPC2, which had a higher number of ASVs relative to their percentage of reads and a higher number of ASVs in Mazard_2012 (Table S2).

The relative abundance of the major subclades followed a similar pattern across amplification methods and cycles, with notable exceptions for subclades IVa and IVb in ST1 and SA2 cycles, respectively. Subclade IVa was dominant in ST1 samples with the Mazard_2012 approach, while the same samples processed with Ong_2022 showed similar contributions of subclades IVa and Ib to the total community (Fig. 1; Table S3). In cycles ST2 and SA1, the proportions of subclade IVa decreased and subclade IVb increased in both methods. In SA2 and SA3, subclade IVb became dominant only in Mazard_2012 samples (Fig. 1; Table S3). Clades CRD1, EnvA, and EnvB were found in higher proportions in subantarctic oceanic cycles SA2 and SA3 than in more coastal SA1 and oceanic subtropical cycles for both amplifications (Fig. 1; Table S3). The mean percentages of reads for each subclade in cycles ST1, ST2, and SA1 were less than 0.004% and 0.06% for Mazard_2012 and Ong_2022, respectively. The mean percentages of reads increased in cycles SA2 and SA3 to 0.06% and 2.8% for CRD1, 0.003% and 0.58% for EnvA, and 0.09% and 0.35% for EnvB for Mazard_2012 and Ong_2022, respectively.

### ASV sequence similarity to reference database.

**FIG 3:**
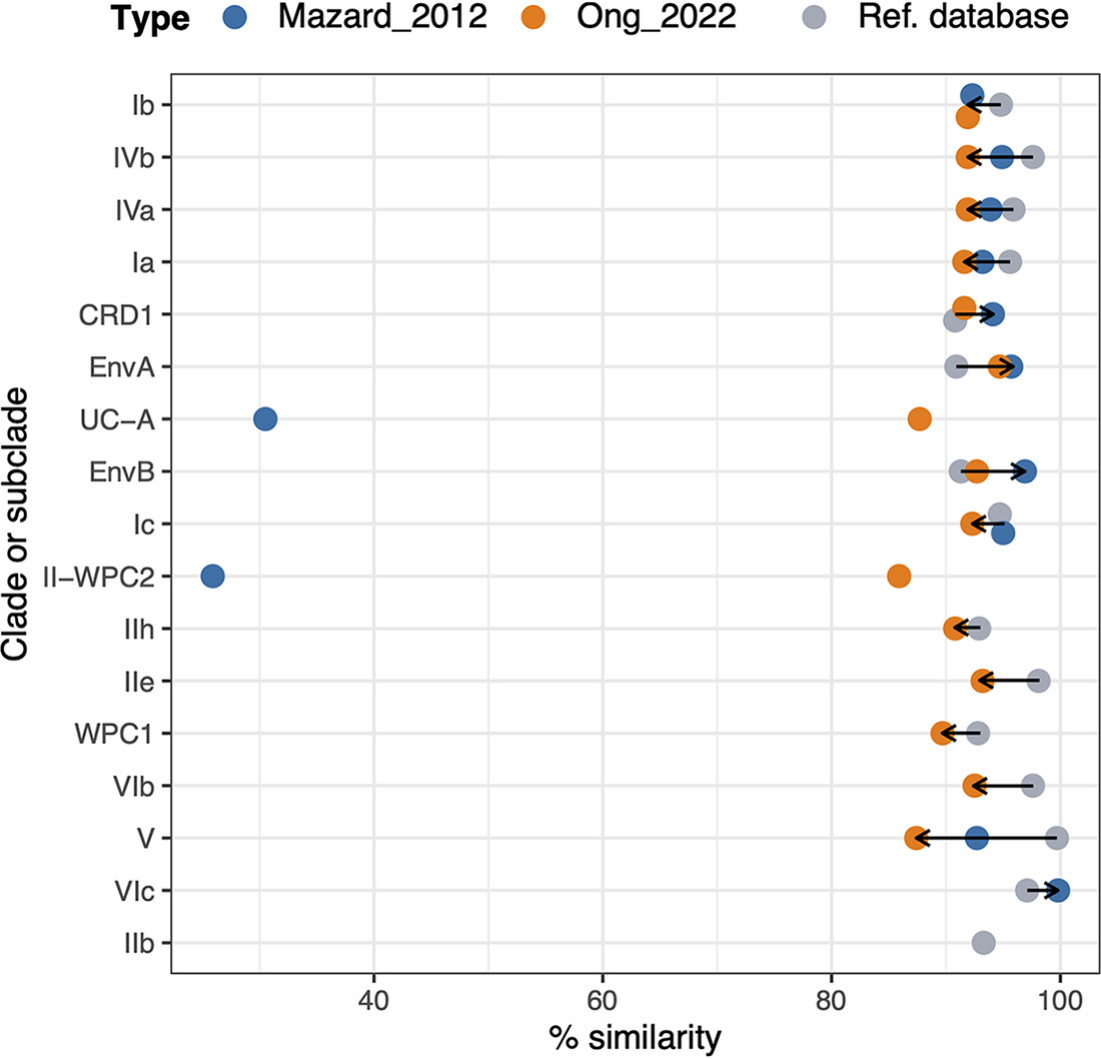
ASV nucleotide similarity within each clade and subclade obtained with the Mazard_2012 and Ong_2022 approaches and the reference sequence database.

Within each clade and subclade, we compared the nucleotide similarities of the ASVs obtained with Mazard_2012 and Ong_2022 and with the reference sequences in the database (32) (Fig. 3; Table S4). In general, similarity between the ASVs within each clade and subclade obtained with both PCR approaches was lower than the reference database, showing that both primer sets were able to capture a higher diversity than the one available in the database (Fig. 3). The only exceptions were clades CRD1, EnvA, and EnvB and subclade VIc (Fig. 3; Table S4). Amid the clades and subclades amplified by both PCR approaches, percentages of similarity within the ASVs obtained with Ong_2022 (from 85.9% to 94.7%) were lower than those obtained with Mazard_2012 (from 92.3% to 99.8%) for all the subclades, with two exceptions, clade UC-A (87.7% and 30.5%, respectively) and subclade II-WPC2 (85.9% and 25.9%, respectively) (Fig. 3; Table S4), indicating that Ong_2022 captured a higher microdiversity than Mazard_2012.

To confirm the taxonomic assignment of the ASVs, the nucleotide sequences of ASVs and references were translated into protein sequences. For each clade and subclade, we compared the translated ASVs from each PCR approach (Mazard_2012 and Ong_2022) against those of the translated reference sequences. ASVs for which the protein sequence had 95% pairwise identity against translated reference sequences were regarded as correctly assigned. Overall, Ong_2022 had a higher proportion of total ASVs that were correctly assigned than did Mazard_2012 (98.2% versus 84.2%) (Table S4). The percentages of correctly assigned ASVs in each clade and subclade were comparable between Mazard_2012 (from 96.2% to 100%) and Ong_2022 (from 90.5% to 100%), except for clade UC-A (no correctly assigned ASVs) and subclade II-WPC2 (2.4%) obtained with Mazard_2012 (Table S4). This result shows that several ASVs in Mazard_2012 assigned to UC-A and II-WPC2 did not represent *petB* gene sequences, and hence the very low percentage of nucleotide similarity among the ASVs for these lineages as mentioned previously. These ASVs assigned to UC-A and II-WPC2 from Mazard_2012 amplification were further examined by BLAST against the GenBank nucleotide database. More than half of these ASVs had no sequence match in the database, indicating that they could have resulted from spurious amplification or that the current database did not have a corresponding reference sequence.

### Community composition from flow cytometry-sorted *Synechococcus* populations.

**FIG 4:**
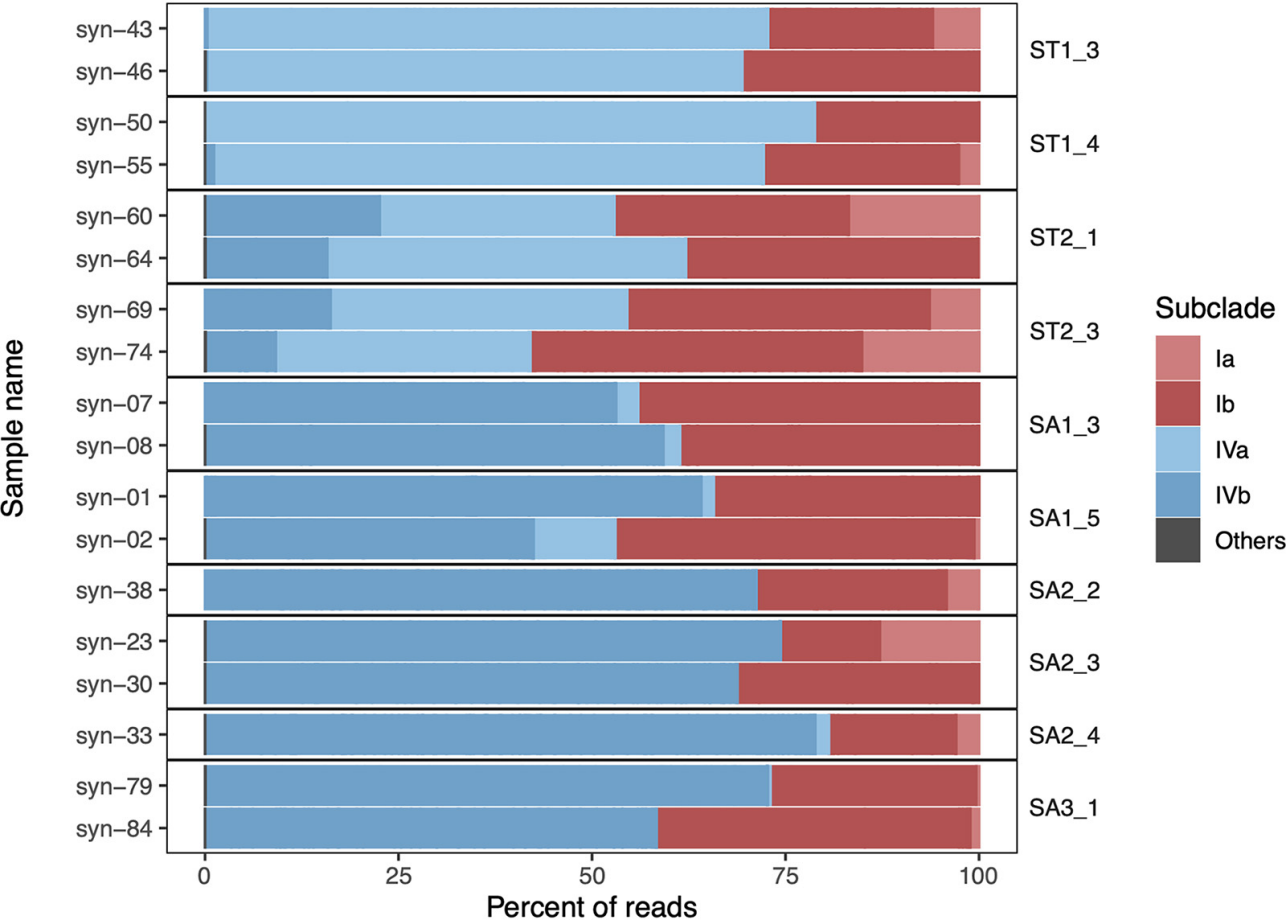
*Synechococcus* taxonomic composition at clade and subclade level from flow cytometry-sorted populations. Samples were grouped by cycle and ordered across a spatial gradient, from subtropical (ST) to subantarctic (SA) cycles. The SUR and DCM depth samples were combined.

We tested our nested PCR with *Synechococcus* cells sorted by flow cytometry from 18 water samples collected in five cycles (Table S1). The 998 ASVs obtained from sorted cells with Ong_2022 were assigned to fewer clades and subclades belonging to subcluster (SC) 5.1 than the ASVs obtained from filtered samples (Fig. 4; Fig. S5).

Similar to the results obtained with filtered samples, Ia, Ib, IVa, and IVb were the major subclades present in all the samples, representing an average of 99.98% of total reads (Fig. 4; Table S5). Clades UC-A, CRD1, and EnvB and subclade II-WPC2 had low contributions to the total community (Fig. 4; Table S5). The mean percentages of reads for subclades Ia and Ib (3.84% and 31.33%, respectively) from sorted cells were lower than those obtained from filtered samples (8.51% and 46.73%) (Tables S2 and S5). In contrast, for subclades IVa and IVb, the mean percentages of reads were higher (25.47% and 39.35%, respectively) in sorted cells than in filtered samples (11.96% and 31.40%) (Table S2 and S5).

Among minor clades and subclades, clade UC-A was found in 11 of 18 sorted samples with an average proportion of reads of 0.01% in contrast with filtered samples, for which this clade represented 0.13% of reads and was present in all samples. Clades CRD1 and EnvB and subclade II-WPC2 were present in 1 or 2 sorted samples, while in filtered samples, they were present in one-third to one-half of the samples. Clades CRD1 and EnvB and subclade II-WPC2 had lower average percentages of reads (0.003%, 0.002%, and 0.002%) than those in Ong_2022 (0.93%, 0.11%, and 0.01%). The 7 minor clades and subclades (EnvA, WPC1, V, IIh, IIe, Ic, and VIb) that were found in filtered samples were not present in sorted samples.

The average number of ASVs per sample obtained from sorted *Synechococcus* cells was almost 2.5 times lower than that from filtered samples (82 ± 35 ASVs and 217 ± 63 ASVs, respectively). Each clade and subclade also had a lower number of ASVs. The relative abundance of major subclades followed a similar pattern in sorted *Synechococcus* and filtered seawater samples, with the exceptions of subclades IVa and IVb (Fig. 1 and 4). Subclade IVa and subclade IVb were dominant in ST and SA sorted *Synechococcus* populations, respectively. This pattern was also observed with the Mazard_2012 approach on filtered samples but differed with the Ong_2022 approach, where the overall contribution of subclades IVa and IVb was similar to that of subclades Ia and Ib (Fig. 1).

## DISCUSSION

We have established a nested PCR protocol to assess the diversity of marine *Synechococcus* populations based on metabarcoding analysis of the *petB* gene marker. Our protocol combines a pair of newly developed primers (petB-50F and petB-634R) and the original primer set (petB-F and petB-R) established by Mazard et al. (12). We have compared the sensitivity and specificity of the new nested amplification protocol (Ong_2022) against those of the original standard PCR (Mazard_2012) on DNA extracted from filtered seawater samples and determined the performance of the proposed nested amplification protocol with flow cytometry-sorted *Synechococcus* populations. While both standard and nested PCRs worked with nearly all filtered seawater samples, sorted *Synechococcus* samples could not be amplified with the Mazard_2012 standard PCR, likely due to a low initial concentration of *Synechococcus* DNA. Nested PCR is often employed when the DNA concentration is too low to be amplified by a standard PCR (37, 38), and thus the Ong_2022 approach could be an alternative method when the initial concentration of *Synechococcus* cells is expected to be low, especially in sorted samples.

None of the ASVs obtained by the two methods using filtered samples have been assigned to *Prochlorococcus*, although *Prochlorococcus* cells were detected by flow cytometry (from 0.3 × 10^3^ to 90.6 × 10^3^ cells mL^−1^) in subantarctic water samples (SA1, SA2, and SA3) (data not shown). Similar to our new primers, petB-F and petB-R of Mazard et al. (12) are also biased toward *Synechococcus* (12). However, during their study, *Prochlorococcus petB* sequences were recovered at low frequency from samples where *Prochorococcus* cell abundances were at least 45 times higher than those of *Synechococcus*. In our study, *Synechococcus* cells were always at the same or higher abundance (1.2 to 45 times) than *Procholorococcus*, which might explain the absence of ASVs assigned to the latter.

ASVs assigned to *Synechococcus* SC 5.2 and SC 5.3 were also not detected. The primers of Mazard et al. (12) (petB-F and petB-R) and our primers (petB-50F and petB-634R) are biased toward SC 5.1, which is represented in the database by a majority of the sequences (32). In addition, *Synechococcus* cells from SC 5.2 and SC 5.3 were probably absent in our samples. SC 5.2 representatives are mainly found in estuaries and river-influenced coastal waters (39–42), and although SC 5.3 contains both freshwater and marine representatives (32, 43), the latter have been sporadically detected at high abundance only in some regions, such as the Red Sea, the Mediterranean Sea, and the northwestern Atlantic Ocean (32, 44, 45).

One of the main concerns with nested PCR is the generation of chimeric sequences that are not true members of the community (37). Indeed, based on chimera removal analysis, our nested PCR approach had twice the proportion of chimeras detected than that detected by the standard amplification. However, Ong_2022 had an overall higher proportion of correctly assigned ASVs than Mazard_2012, indicating that the nested PCR was more specific for *Synechococcus*. While the use of a higher number of cycles might increase the chimera generation and sensitivity (e.g., Ong_2022 generally increased the proportion of reads of low-abundance clades and subclades) in nested approaches, the improved specificity of Ong_2022 PCR is derived from the binding of two separate sets of oligonucleotides to the same target template (46). Additionally, Ong_2022 PCR captured a higher genetic diversity of *Synechococcus* SC 5.1 than Mazard_2012 PCR. Within common subclades amplified by both Mazard_2012 and Ong_2022 PCRs, almost all subclades had a higher number of ASVs in the latter approach. These ASVs had lower nucleotide similarity within each subclade, indicating that our approach was able to capture a higher microdiversity within *Synechococcus* subclades. This directly contrasts with previous studies, which reported that nested PCR approaches resulted in a lower number of lineages and operational taxonomic units than a standard PCR (37, 38).

The number and proportion of reads of minor clades and subclades decreased in sorted samples (11 versus 4 clades and subclades, 1% versus 0.2% of reads for filtered and sorted samples, respectively). Metz et al. (19) investigated the diversity of photosynthetic picoeukaryotes in lakes using a similar approach. They found that the richness of filtered samples was on average lower than that in the sorted samples. One major difference between the sorting protocols of Metz et al. (19) and Ong_2022 is the number of cells sorted. In the Metz et al. (19) protocol, the number of cells sorted was 22 to 50 times higher than that in the Ong_2022 protocol. Since microbial plankton communities contain a large number of taxa that are present in low abundance (47), a larger sample size would probably increase the number of taxa detected and improve the representation of rare taxa with the Ong_2022 approach for sorted cells (48). However, the trade-off between flow cytometry sorting time and the detection of rare microbial groups needs to be considered when designing the study. Cell sorting by flow cytometry is a time-consuming process, and increasing the number of cells sorted for numerous natural marine samples might not be feasible.

The taxonomic diversity obtained with our primers agreed with the clade distribution observed in previous studies conducted with other marker genes and PCR-free methods in geographical regions with similar environmental conditions. For example, subclades Ia, Ib, IVa, and IVb were the major lineages observed in the data sets obtained from filtered samples (~99%) and sorted samples (99.8%) with nested and standard PCRs. This agrees with previous reports in which clades I and IV most often co-occur in the open ocean at latitudes above 30°N and below 30°S (9, 12, 16, 32, 45, 49). However, the relative abundance of clades I and IV varied between filtered samples amplified by Mazard_2012 and Ong_2022 PCRs and sorted samples. Filtered samples amplified with Mazard_2012 and sorted samples amplified with Ong_2022 followed a similar pattern, whereby clade IV dominated in subtropical and subantarctic waters. In contrast, the proportion of clades I and IV remained similar across sampling locations when filtered samples were amplified with Ong_2022. Prior studies conducted in surrounding areas report different proportions of clades I to IV. Both clades were detected in similar proportions along the coast of New Zealand at overlapping latitudes (45°S) by a PCR-independent method (42). Clade IV has been observed in a higher proportion than clade I in the western and eastern South Pacific Ocean at slightly lower latitudes with warmer conditions (around 30°S compared to 42°S in this study) (9, 32, 45). Hence, we cannot ascertain which method more closely reflects the natural abundance of the dominant clades.

We observed that relative abundances of clades CRD1, EnvA, and EnvB increased in high-nutrient, low-chlorophyll (HNLC) subantarctic waters where iron is typically the proximate limiting nutrient (filtered samples amplified by both approaches) (50, 51). Clade CRD1 was reported to cooccur with clades I and IV in the southeastern Pacific Ocean and other cold and high-latitude HNLC conditions (16, 32, 45). More recent reports suggested that EnvA and EnvB also correspond to lineages found under low-iron conditions (32, 45, 52, 53). The Ong_2022 approach recorded subclades IIh and IIe in cycle ST1 at a very low abundance. Although members from clade II are mostly warm thermal types that dominate in nutrient-depleted tropical areas (3), subclades IIe and IIh were also reported at low relative abundances in cooler waters (14.1 to 17.5°C), where iron was not limiting for phytoplankton growth (32). Surface waters at ST1 were at a similar temperature at the time of sampling (13°C), which is likely within the temperature range where subclades IIe and IIh were detected. Subclades WPC1, VIb, VIc, and V were detected with low relative abundances, but their precise ecological niche and biogeographical distribution have not yet been established (32).

In summary, we have designed a nested PCR protocol with two new primer sequences to amplify the *petB* gene from marine *Synechococcus* populations. Results obtained from filtered seawater samples suggest that the new nested PCR approach is specific for *Synechococcus* and captured a wider genetic diversity, especially for rare groups. The nested PCR protocol successfully amplified *Synechococcus* cells from flow cytometry-sorted samples and recovered a composition of the dominant subclades similar to that of filtered samples. Our amplification protocol will improve our understanding of *Synechococcus* communities by allowing the determination of *Synechococcus* diversity directly associated with quantitative measurements (e.g., nutrient cell ratios or carbon update rates) obtained using sorted samples.

## MATERIALS AND METHODS

### Sampling location.

Samples were collected during the Salp Particle expOrt and Oceanic Production (SalpPOOP) cruise (TAN1810, 21 October to 21 November 2018) conducted near the Chatham Rise (Aotearoa-New Zealand) on board the R/V *Tangaroa*. The Chatham Rise is a dynamic region where northward-moving subtropical (ST) water masses mix with southward-moving subantarctic (SA) water masses to form the Subtropical Convergence Zone (54–57). ST waters are warm, saline, and macronutrient depleted, while SA waters are cool, less-saline, and high-nitrate, low-chlorophyll, and low-silicate (HNLC-LSi) waters where low iron and silicate are the primary limiting factors for phytoplankton growth and productivity (50, 58).

We adopted a Lagrangian sampling strategy using a satellite-tracked 15-m drogue drifted array that allowed us to track and sample the same water parcel multiple times over 3 to 6 days (here referred to as “cycles”) (59). Five cycles were conducted, two in subtropical waters (ST1 and ST2) and three in subantarctic waters (SA1, SA2, and SA3) (see Fig. S1 in the supplemental material). In each cycle, water column profiles of temperature, salinity, dissolved oxygen, photosynthetically active radiation (PAR), and fluorescence were measured 4 to 6 times daily by a Seabird (SBE 911plus) conductivity-temperature-depth (CTD) instrument with a PAR sensor. The cycles were identified as subtropical or subantarctic based on physical measurements during sampling in accordance with the definitions of Lüskow et al. (57). Samples used for picocyanobacterial community analysis presented in this study were collected using a CTD rosette sampler equipped with 10-L Niskin bottles from six depths (5 to 70 m). For DNA analysis of filtered seawater samples, 1.5 to 2 L of seawater from all depths was filtered through a 0.22-μm-pore-size Sterivex filter using a peristaltic pump (number of samples = 71) (Table S1). For DNA analysis of flow cytometry-sorted samples, 1.5-mL samples were collected from the surface mixed layer (SUR; 12 m) and the deep chlorophyll maximum (DCM; 40 or 70 m) (Table S1), where they were preserved with a solution of dimethyl sulfoxide (DMSO) and 1/100 Pluronic acid mix at a 10% final concentration and flash-frozen in liquid nitrogen (number of samples = 21). All samples were stored at –80°C until laboratory processing.

### Flow cytometry cell sorting.

*Synechococcus* cells were sorted using a FACSAria flow cytometer (Becton, Dickinson, San Jose, CA), equipped with a laser emitting at 488 nm, a 70-mm nozzle, and the following filters: 488/10 band pass (BP) for size side scatter (SSC), 576/26 BP for orange fluorescence, and 655 long pass for red fluorescence. *Synechococcus* populations were detected by their signature in plots of chlorophyll *a* red fluorescence versus SSC and discriminated from photosynthetic picoeukaryotes based on the orange fluorescence of phycoerythrin. Filter-sterilized (0.22-μm pore size) Tris-HCl (50 mM, pH 8.0)–NaCl (10 mM) buffer was used as the sheath liquid. Sheath pressure was set at 70 lb/in^2^, and nozzle frequency was 90,000 Hz with a deflection voltage of 6,000 V. Cells were sorted in purity mode and collected into Eppendorf tubes containing Tris-EDTA lysis buffer (Tris [10 mM], EDTA [1 mM], and 1.2% Triton, final concentration) as described previously (18). Sheath fluid samples collected during sorting were processed and analyzed as negative controls in all subsequent steps, including sequencing, to test for contamination in the flow sorting process.

### petB-50F and petB-634R primer design.

The criteria for primer design were as follows: (i) 3 or fewer mismatches in the binding region, (ii) no nucleotide ambiguity or mismatches at the 5′ and 3′ ends, (iii) low degeneracy, (iv) binding region between 15 and 20 bp, (v) annealing temperature difference between the forward and reverse primers not greater than 4°C, (vi) insertions and deletions among the different groups should be absent from the primer binding region, and (vii) no hairpins and self-dimerization within the primer sequence. Four hundred seven *Synechococcus* nucleotide sequences of the *petB* gene from the Farrant et al. (32) reference database were aligned using Multiple Alignment and Fast Fourier Transform (MAFFT) alignment version 7.450 (60) in Geneious Prime version 2019.2.3 (61) with default parameters and used to locate the binding region conforming to the criteria described. The selected forward primer petB-50F is located at positions 50 to 66 with reference to the first nucleotide of the *petB* gene from *Synechococcus* sp. WH8109 (accession number CP006882) (Table 1). The selected reverse primer, petB-634R, is located at positions 615 to 634 with reference to the first nucleotide of the *petB* gene from *Synechococcus* sp. WH8109 (accession number CP006882) (Table 1). Primer petB-50F had no mismatches in the binding region for *Synechococcus* SC 5.1 reference sequences, 2 to 3 mismatches for SC 5.2 and SC 5.3, and excluded *Prochlorococcus* (≥3 mismatches) and other marine cyanobacteria (e.g., *Richelia* sp.). Primer petB-634R had mainly no mismatches in the binding region for *Synechococcus* SC 5.1 to SC 5.3 and excluded *Prochlorococcus* (≥3 mismatches). After the binding region was selected, the sequences were truncated to the length amplified by the new forward primer sequence and petB-R of Mazard et al. (12) and used to infer a maximum likelihood phylogenetic tree with FastTree version 2.1.11 (62) in Geneious Prime with default parameters. The phylogenetic tree was used to determine if the shorter *petB* amplicon could distinguish between subclades. All alignments and phylogenetic trees can be found in https://github.com/deniseong/marine-Synechococcus-metaB.

### DNA extraction and PCR.

DNA from Sterivex filters was extracted using the Nucleospin plant II DNA extraction kit from Macherey-Nagel with modification of the manufacturer’s instructions. The modified protocol can be found at https://github.com/deniseong/marine-Synechococcus-metaB. DNA from flow cytometry-sorted *Synechococcus* cells was obtained using three cycles of flash-freezing and thawing in liquid nitrogen (18). The Mazard_2012 protocol refers to PCR amplification with petB-F (5′-TACGACTGGTTCCAGGAACG-3′) and petB-R (5′-GAAGTGCATGAGCATGAA-3′) primers (12), performed on extracted DNA from filtered seawater. PCRs were done using the following reaction mixture: extracted DNA template with an average final concentration of 1.3 ng μL^−1^ (from 0.007 to 4.11 ng μL^−1^), 12.5 μL KAPA HiFi hotstart readymix 2×, 0.3 μM primers (petB-F and petB-R) (final concentration), 0.1 to 0.4 μg μL^−1^ final concentration of bovine serum albumin (BSA), and H_2_O to a 25-μL final reaction volume. Thermal conditions were as follows: 94°C for 5 min, followed by 30 cycles of 94°C for 30 s, 55°C for 30 s, and 72°C for 45 s, and a final cycle of 72°C for 6 min. All PCRs were performed in duplicate, and results were pooled. The Ong_2022 protocol refers to a nested PCR performed on extracted DNA from either filtered seawater or flow cytometry-sorted *Synechococcus* cells. The first round of PCR amplification was done using the following reaction: extracted DNA template with an average final concentration of 0.54 ng μL^−1^ (from 0.015 to 2.055 ng μL^−1^) or a volume corresponding to approximately 160 to 400 sorted *Synechococcus* cells (4 μL maximum), 5 μL KAPA HiFi hotstart readymix 2×, 0.3 μM final concentration of primer petB-F, 0.3 μM final concentration of primer petB-634R (5′-GCTTVCGRATCATCARGAAG-3′), 0.1 μg μL^−1^ final concentration of BSA (only for filtered samples), and H_2_O for a 10-μL reaction volume. Thermal conditions were as follows: 94°C for 5 min, followed by 30 cycles of 94°C for 30 s, 59°C for 30 s, and 72°C for 45 s, and a final cycle of 72°C for 6 min. For the second round of amplification, the following conditions were used: 2.5 μL of first-round product, 12.5 μL KAPA HiFi hotstart readymix 2×, 0.3 μM final concentration of primer petB-50F (5′- TYCAGGACATYGCTGAY-3′), 0.3 μM final concentration of primer petB-R, and H_2_O for a 25-μL reaction volume. Thermal conditions were as follows: 94°C for 5 min, followed by 30 cycles of 94°C for 30 s, 55°C for 30 s, and 72°C for 45 s, and a final cycle of 72°C for 6 min. All first- and second-round PCRs were performed in duplicate, and results were pooled. Samples were purified, barcoded, and sequenced by the GeT-PlaGe platform of GenoToul (INRAE, Auzeville, France) using an Illumina MiSeq platform (2 × 300 bp).

### *petB* rRNA gene amplicon analysis.

Sequences were processed on RStudio version 1.4.1717 (63). Primer sequences were removed using Cutadapt version 3.4 (64). Fastq files were trimmed and quality filtered using the function ‘filterandtrim’ with the DADA2 R package version 1.12 (65). Based on sequence quality score, reads obtained with the Mazard_2012 and Ong_2022 methods were trimmed and filtered using the following options: truncLen = c(280, 280) and c(250, 280), minLen = c(280, 280), and minLen = c(250, 280), respectively, with truncQ = 2 and maxEE = c(10, 10) in both data sets. Forward and reverse sequences were dereplicated and grouped. To merge the forward and reverse reads, the ‘mergePairs’ function on DADA2 was used with the justConcatenate = TRUE option to add 10 degenerate base ‘N’ (for any one base) between forward and reverse reads. Chimeras were identified and removed using the function removeBimeraDenovo. Taxonomy was obtained with the function ‘assignTaxonomy’. ASVs were assigned against the *petB* reference database of Farrant et al. (32) reformatted for use with DADA2. The following taxonomic nomenclature was maintained: three major *Synechococcus/Cyanobium* lineages called subclusters (SC) 5.1 through 5.3, 14 clades (I to IX, XVI, XX, UC-A, WPC1, CRD1, EnvA, and EnvB) within SC 5.1, and 20 subclades (Ia to Ic, IIa to IIh, and II-WPC2, IIIa to IIIc, IVa and IVb, and VIa to VIc). ASVs assigned as *Richelia* sp. were removed prior to normalization of reads. At station SA1_5, sorted samples from two depths (12 m and 40 m) were sorted to two and three different total numbers of cells (Table S1). As the communities were similar at each depth, only samples that had 10,000 cells sorted were used for analysis, and for filtered samples, all depths were combined. Analysis was performed with R Phyloseq package version 1.36.0 (66) and Geneious Prime version 2019.2.3 (61). All sequence processing and analysis of scripts along with the reformatted database can be found at https://github.com/deniseong/marine-Synechococcus-metaB.

### Data availability.

Raw sequencing data were deposited in the NCBI Sequence Read Archive under BioProject number PRJNA885274. Source code, DNA extraction protocol, and all metadata generated are available on GitHub (https://github.com/deniseong/marine-Synechococcus-metaB). Raw data files from flow cyyometry sorting are available at http://flowrepository.org/id/FR-FCM-Z5P8 (repository ID, FR-FCM-Z5P8). Step-by-step protocols for DNA extraction and PCR amplification are published on protocols.io at https://doi.org/10.17504/protocols.io.5qpvorkj7v4o/v1.

## Supplementary Material

Reviewer comments
