# Supplementary material for: Nested PCR Approach for petB Gene Metabarcoding of Marine Synechococcus Populations
Source: Microbiol Spectr. 2023 Mar 6;11(2):e04086-22. doi: 10.1128/spectrum.04086-22 (PMC10100653; doi:10.1128/spectrum.04086-22)
Supplement: Supplemental file 1 — Supplemental material. Download spectrum.04086-22-s0001.pdf, PDF file, 0.8 MB [file spectrum.04086-22-s0001.pdf]

SUPPLEMENTAL MATERIAL

List of Figures

Figure S1 Study area with cycle locations at the Chatham Rise east of Aotearoa-New Zealand. Black dots represent the sampling location within each cycle. Bathymetric lines (m) are indicated. Bathymetry was obtained from Amante & Eakins (1) and plotted with ggOceanMaps (2). Sea surface temperature data (°C) was obtained from MODIS (NASA) and averaged over November 2018. ST and SA refers to subtropical and subantarctic respectively and numbers to the cycle 2

Figure S2 *Synechococcus* taxonomic composition at clade and subclade level using Mazard_2012 and Ong_2022 protocol on filtered seawater samples and sorted *Synechococcus* cells. Samples were grouped by station. Each station was labelled as 'cycle'_'station' and ordered across a spatial gradient, from subtropical (ST) to subantarctic (SA) cycles. . . 3

List of Tables

Table S1 Location and date of samples.

Table S2 Number of ASVs and percentage of reads at clade and subclade level for 70 distinct samples: mean, maximum, minimum and standard deviation, and the total number of ASVs using Mazard_2012 and Ong_2022. Significant differences of the percent of reads per clade or subclade between the two approaches were tested using a paired sample Wilcoxon test (P-value < 0.05). The rows are arranged in descending order according to the mean percentage of reads from Ong_2022.

Table S3 The mean percentage of reads for each clade or subclade in each cycle, using Mazard_2012 and Ong_2022 amplification protocol. The rows are arranged in descending order according to the mean percentage of reads of each clade or subclade from Ong_2022.

Table S4 Percent similarity of nucleotide sequences/ASVs and percent of correctly assigned ASVs in each clade or subclade from Mazard_2012 and Ong_2022 approaches on filtered samples and reference database.

Table S5 Percentage of reads at clade and subclade level across 18 samples: mean, maximum, minimum and standard deviation, and the total number of ASVs of sorted *Synechococcus* cells. The rows are arranged in descending order according to the mean percentage of reads.

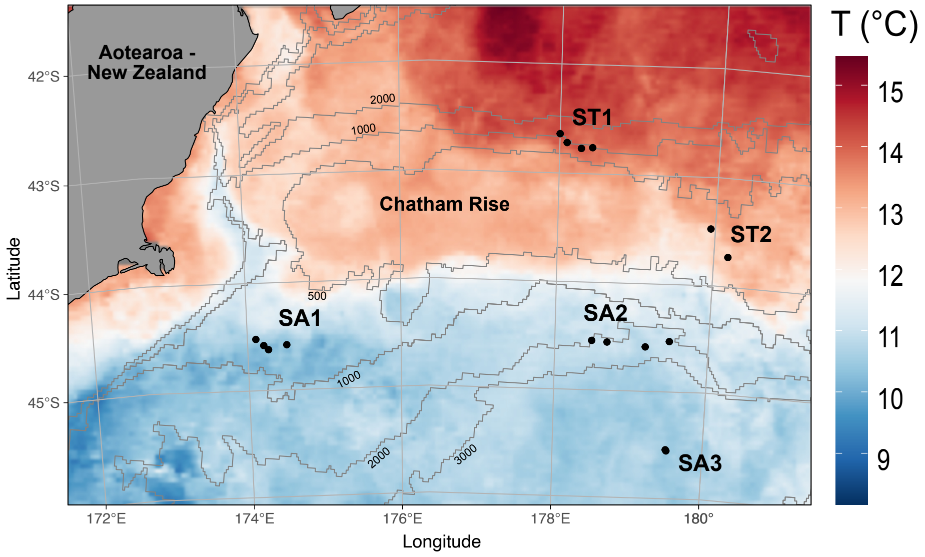

FIG S1 **Study area with cycle locations at the Chatham Rise east of Aotearoa-New Zealand. Black dots represent the sampling location within each cycle. Bathymetric lines (m) are indicated. Bathymetry was obtained from Amante & Eakins (1) and plotted with ggOceanMaps (2). Sea surface temperature data (°C) was obtained from MODIS (NASA) and averaged over November 2018. ST and SA refers to subtropical and sub-antarctic respectively and numbers to the cycle**

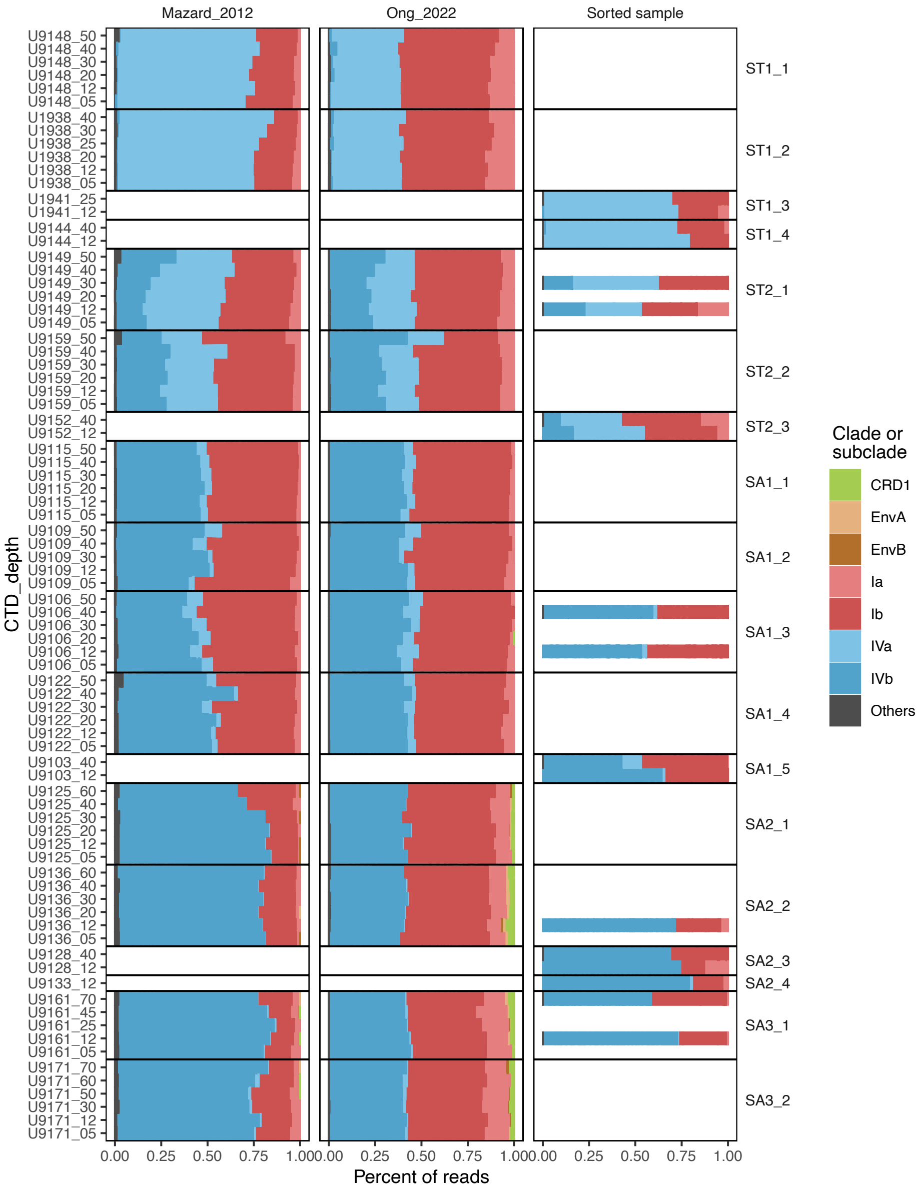

FIG S2 ***Synechococcus* taxonomic composition at clade and subclade level using Mazard_2012 and Ong_2022 protocol on filtered seawater samples and sorted *Synechococcus* cells. Samples were grouped by station. Each station was labelled as 'cycle'_'station' and ordered across a spatial gradient, from subtropical (ST) to subantarctic (SA) cycles.**

REFERENCES

1. **Amante C, Eakins BW**. ETOPO1 1 Arc-Minute Global Relief Model Procedures, Data Sources and Analysis .

2. **Vihtakari M**. 2022. ggOceanMaps: Plot Data on Oceanographic Maps using 'ggplot2'. https://CRAN.R-project.org/package=ggOceanMaps. R package version 1.2.6.
