# Supplementary material for: Nested PCR Approach for petB Gene Metabarcoding of Marine Synechococcus Populations
Source: Microbiol Spectr. 2023 Mar 6;11(2):e04086-22. doi: 10.1128/spectrum.04086-22 (PMC10100653; doi:10.1128/spectrum.04086-22)
Supplement: Reviewer comments [file reviewer-comments.pdf]

AMERICAN SOCIETY FOR MICROBIOLOGY | Microbiology Spectrum

Nested PCR approach for *petB* gene metabarcoding of marine *Synechococcus*

Denise Rui Ying Ong, Andrés Gutiérrez-Rodríguez, Laurence Garczarek, Dominique Marie, and Adriana Lopes dos Santos

Corresponding Author(s): Denise Rui Ying Ong, Nanyang Technological University

Review Timeline:

Submission Date:	October 14, 2022
Editorial Decision:	November 8, 2022
Revision Received:	January 30, 2023
Accepted:	January 30, 2023

Editor: Blaire Steven

Reviewer(s): The reviewers have opted to remain anonymous.

Transaction Report:

(Note: With the exception of the correction of typographical or spelling errors that could be a source of ambiguity, letters and reports are not edited. The original formatting of letters and referee reports may not be reflected in this compilation.)

DOI: https://doi.org/10.1128/spectrum.04086-22

November 8, 2022

Mx. Denise Rui Ying Ong
Nanyang Technological University
50 Nanyang Avenue
SIngapore
Singapore

Re: Spectrum04086-22 (Nested PCR approach for *petB* gene metabarcoding of marine *Synechococcus*)

Dear Mx. Denise Rui Ying Ong:

Please see the comments of reviewer below. As editor I would also add that more methodological detail should be added to a paper that describes a new method in order to foster further adoption.

Thank you for submitting your manuscript to Microbiology Spectrum. When submitting the revised version of your paper, please provide (1) point-by-point responses to the issues raised by the reviewers as file type "Response to Reviewers," not in your cover letter, and (2) a PDF file that indicates the changes from the original submission (by highlighting or underlining the changes) as file type "Marked Up Manuscript - For Review Only". Please use this link to submit your revised manuscript - we strongly recommend that you submit your paper within the next 60 days or reach out to me. Detailed instructions on submitting your revised paper are below.

Link Not Available

Below you will find instructions from the Microbiology Spectrum editorial office and comments generated during the review.

ASM policy requires that data be available to the public upon online posting of the article, so please verify all links to sequence records, if present, and make sure that each number retrieves the full record of the data. If a new accession number is not linked or a link is broken, provide production staff with the correct URL for the record. If the accession numbers for new data are not publicly accessible before the expected online posting of the article, publication of your article may be delayed; please contact the ASM production staff immediately with the expected release date.

The ASM Journals program strives for constant improvement in our submission and publication process. Please tell us how we can improve your experience by taking this quick Author Survey.

Sincerely,

Blaire Steven

Editor, Microbiology Spectrum

Journals Department
American Society for Microbiology
1752 N St., NW
Washington, DC 20036
E-mail: spectrum@asmusa.org

Reviewer comments:

Reviewer #1 (Comments for the Author):

Review for: Nested PCR approach for petB gene metabarcoding of marine Synechococcus

This study is based on the use of the single copy petB gene (a subunit of cytochrome B6f) as a marker to for sequence-based comparisons of diversity among Synechococcus spp. The paper outlines a metabarcoding approach to be used alongside flow cytometry-based cell sorting and presents its applications studying the diversity of marine phytoplankton alongside cell physiology measurements.

One basis of this study is to present an alternative to 16S rRNA gene amplicon approaches by targeting genes that may provide better resolution between closely related Synechococcus spp. The authors outline the application of nested PCR that involves multiple sequential amplification steps with different primers to increase sensitivity to low concentrations of template (e.g., samples obtained from cell sorting).

One major point of concern is with respect to article type. It reads as a Methods comparison report and less so as a Research article. For example, the first section of Results is titled: Primer Design and PCR Amplification and the first three figures (out of four) are used to establish differences between protocols.

Along these same lines, I feel that this paper is lacking context on the ecological interpretation and comparisons of the subtropical and subantarctic sampling locations. In fact, there is sparse detail presented on these sampling locations in the main text and one must investigate the Supplementary material and Methods section to become familiarized. There is some discussion on the interpretation of how relative proportions of clades might represent different sampling locations, but no data to support these interpretations (e.g., L 371 and the discussion on clade II being indicative of warmer temperature, nutrient deplete environments).

My other major concern is that the authors justify the application of their method with respect to cell sorting because it allows for molecular diversity analyses to coincide with other measurements of cellular physiology such as "nutrient stoichiometric and isotopic composition and cellular activity such as glucose, phosphorus, nitrogen and CO2 uptake rates." However, none of these measurements were demonstrated within this study or related to the metabarcoding methodology being presented. This is a major overreach that extends beyond results supported by the data.

My primary recommendation is that the authors revise this paper to clearly communicate the goal of establishing a methodology or to include more detailed reporting on the diversity and ecological considerations being drawn between the various Synechococcus clades. If the authors wish to revise the paper to be more focused on methodology, then I think the readers would also benefit from more specific materials related to reproducible, step-by-step protocols.

Other major comments:

Abstract:
-first sentence "...cannot be accurately obtained using only 16S rRNA gene given its relatively low sequence divergence between closely related picocyanobacterial groups." - This is a strong statement for leading a paper off because it lacks any context of what the authors consider to be a benchmark of "molecular diversity" or what "accurately" obtaining this would be.

Paragraph from L 77: Nested PCR methods are an alternative to single cell genomic approaches. This paragraph could go further in explaining the comparative advantages and disadvantages of the two approaches rather than simply leaving it at cost savings.

Paragraph from L 102: This paragraph could be improved by outlining some of the risks and disadvantages of nested PCR (i.e., errors induced from double amplification that would manifest as overestimations of diversity at the ASV-level) in the context of how these risks are mitigated and why the pros outweigh the cons. Alternatively, this material could be more clearly articulated in the Discussion.

L162: these "oceanographic patterns" should be cited to establish this line of reasoning

L243-244: This conclusion is not justified because spurious amplification is not the only reason that an ASV will not match a given database. Please put this statement into the context and limitations of databases in general.

Minor comment:
There are several mistakes in the language and grammar that can easily be fixed via revisions. Therefore, I will not go note every instance.

Staff Comments:

Preparing Revision Guidelines
To submit your modified manuscript, log onto the eJP submission site at https://spectrum.msubmit.net/cgi-bin/main.plex. Go to Author Tasks and click the appropriate manuscript title to begin the revision process. The information that you entered when you first submitted the paper will be displayed. Please update the information as necessary. Here are a few examples of required updates that authors must address:

• Point-by-point responses to the issues raised by the reviewers in a file named "Response to Reviewers," NOT IN YOUR COVER LETTER.
• Upload a compare copy of the manuscript (without figures) as a "Marked-Up Manuscript" file.
• Each figure must be uploaded as a separate file, and any multipanel figures must be assembled into one file.
• Manuscript: A .DOC version of the revised manuscript
• Figures: Editable, high-resolution, individual figure files are required at revision, TIFF or EPS files are preferred

For complete guidelines on revision requirements, please see the journal Submission and Review Process requirements at https://journals.asm.org/journal/Spectrum/submission-review-process. **Submissions of a paper that does not conform to Microbiology Spectrum guidelines will delay acceptance of your manuscript.** "

Please return the manuscript within 60 days; if you cannot complete the modification within this time period, please contact me. If you do not wish to modify the manuscript and prefer to submit it to another journal, please notify me of your decision immediately so that the manuscript may be formally withdrawn from consideration by Microbiology Spectrum.

If your manuscript is accepted for publication, you will be contacted separately about payment when the proofs are issued; please follow the instructions in that e-mail. Arrangements for payment must be made before your article is published. For a complete list of **Publication Fees**, including supplemental material costs, please visit ourwebsite.

Corresponding authors may join or renew ASM membership to obtain discounts on publication fees. Need to upgrade your membership level? Please contact Customer Service at Service@asmusa.org.

Thank you for submitting your paper to Microbiology Spectrum.

Answers to referee comments

Dear editor and referee,

We thank the editor and reviewer for their support of our work and their constructive remarks. Please find below our answers to your comments. Reviewer comments are in blue and our answers in black. We have also provided a file showing the differences between the two versions.

Editor Comments to Author

Please see the comments of reviewer below. As editor I would also add that more methodological detail should be added to a paper that describes a new method in order to foster further adoption.

We thank the editor for the suggestion. This manuscript details a nested PCR protocol of *petB* gene for illumina sequencing from *Synechococcus* populations. The protocol can be used with DNA templates generated by traditional filtration methods and flow cytometry sorted populations. To improve the methodology, we have created a step-by-step protocol explaining the DNA extraction protocol used and the PCR amplification from flow cytometry sorted cells which is available on DOI: 10.17504/protocols.io.5qpvorkj7v4o/v1 (Private link for reviewers: https://www.protocols.io/private/2134A97076D211EDB22C0A58A9FEAC02 to be removed before publication). The link can be found at the "Availability of data and materials" section (lines 536-537). A detailed pipeline and all source code for the processing raw Illumina sequences and data analysis are also available on GitHub (https://github.com/deniseong/marine-Synechococcus-metaB) and provided in the section mentioned before. A README.md file is also provided to help the reader to navigate on the GitHub repository. The manuscript therefore concentrates on the results obtained to validate our primers and protocols.

Reviewer Comments to Author:

Reviewer: 1

One basis of this study is to present an alternative to 16S rRNA gene amplicon approaches by targeting genes that may provide better resolution between closely related Synechococcus spp. The authors outline the application of nested PCR that involves multiple sequential amplification steps with different primers to increase sensitivity to low concentrations of template (e.g., samples obtained from cell sorting).

One major point of concern is with respect to article type. It reads as a Methods comparison report and less so as a Research article.

We agree with the reviewer, and thus we have submitted the manuscript as method and protocol type, and not research article. We are not sure why the reviewer missed this information in the system.

Along these same lines, I feel that this paper is lacking context on the ecological interpretation and comparisons of the subtropical and subantarctic sampling locations. In fact, there is sparse detail presented

on these sampling locations in the main text and one must investigate the Supplementary material and Methods section to become familiarized.

We have improved the description of the water masses sampled in the Materials and Methods to provide a better exploration of the environmental conditions in our results and the discussion.

Edited (lines 408–413): "The Chatham Rise is a dynamic region where northward-moving subtropical (ST) water masses mix with southward moving subantarctic (SA) water masses to form the Subtropical Convergence Zone (1, 2, 3, 4). ST waters are warm, saline and macronutrient-depleted while SA waters are cool, less saline and high-nitrate, low chlorophyll, low-silicate (HNLC-LSi) waters where low iron and silicate are the primary limiting factors for phytoplankton growth and productivity (5, 6)."

However, our objective is to provide the description of a PCR amplification protocol from flow cytometry sorted cells for the analysis of the genetic diversity of *Synechococcus* through high-throughput sequencing. We then used a few samples, from contrasting oceanic conditions, to validate our method. Any further exploration or comparison of ecological significance will require a broad dataset than the one provided and a deep discussion into the oceanographic conditions in which the samples were obtained. We believe both points are not within the scope of this methodological manuscript.

There is some discussion on the interpretation of how relative proportions of clades might represent different sampling locations, but no data to support these interpretations (e.g., L 371 and the discussion on clade II being indicative of warmer temperature, nutrient deplete environments).

As mentioned before, for better contextualization of the environmental conditions in which our samples were obtained, we have improved the description of the water masses sampled.

My other major concern is that the authors justify the application of their method with respect to cell sorting because it allows for molecular diversity analyses to coincide with other measurements of cellular physiology such as "nutrient stoichiometric and isotopic composition and cellular activity such as glucose, phosphorus, nitrogen and CO2 uptake rates." However, none of these measurements were demonstrated within this study or related to the metabarcoding methodology being presented. This is a major overreach that extends beyond results supported by the data.

This manuscript details a nested PCR protocol of *petB* gene for metabarcoding of *Synechococcus* populations. The protocol can be used with DNA templates generated by traditional filtration methods and flow cytometry sorted populations. We have suggested that our protocol would be of interested to those applying flow cytometry for cell physiology measurements, such that these studies can also obtain the community composition simultaneously.

As pointed out in the introduction, the literature has several examples of how flow cytometric sorted *Synechococcus* cells have provided important information about their metabolism (7, 8, 9, 10, 11, 12, 13, 10, 11, 14, 15, 16). However, only few of these studies combined quantitative cell measurements with a fine taxonomic identification of the sorted populations by molecular methods. The current method for a fine taxonomic diversity of *Synechococcus petB* gene marker, is metagenome sequencing mining, which requires more DNA and is more expensive (17, 18).

Our protocol provides an alternative to the scientific community. The scope of this methodological

manuscript was to validate the nested PCR protocol and not to perform cell measurements.

My primary recommendation is that the authors revise this paper to clearly communicate the goal of establishing a methodology or to include more detailed reporting on the diversity and ecological considerations being drawn between the various Synechococcus clades. If the authors wish to revise the paper to be more focused on methodology, then I think the readers would also benefit from more specific materials related to reproducible, step-by-step protocols.

We thank the reviewer for this suggestion. We have created a step-by-step protocol for DNA extraction and PCR amplification, published on protocols.io at DOI: 10.17504/protocols.io.5qpvorkj7v4o/v1 (Private link for reviewers: https://www.protocols.io/private/2134A97076D211EDB22C0A58A9FEAC02 to be removed before publication) and added to the "Availability of data and materials" section (lines 536-537). A detailed pipeline and all source code for processing raw Illumina sequences and data analysis are available on GitHub (https://github.com/deniseong/marine-Synechococcus-metaB).

Other major comments:

Abstract: -first sentence "...cannot be accurately obtained using only 16S rRNA gene given its relatively low sequence divergence between closely related picocyanobacterial groups." - This is a strong statement for leading a paper off because it lacks any context of what the authors consider to be a benchmark of "molecular diversity" or what "accurately" obtaining this would be.

We have changed the statement to compare the use of high-resolution marker genes against 16S rRNA gene.

Original: "The molecular diversity of marine picocyanobacterial populations, an important component of phytoplankton communities, cannot be accurately obtained using only 16S rRNA gene given its relatively low sequence divergence between closely related picocyanobacteria groups."

Edited (Lines 11-15): "The molecular diversity of marine picocyanobacterial populations, an important component of phytoplankton communities, is better characterised using high-resolution marker genes compared to the 16S rRNA gene as it has greater sequence divergence to differentiate between closely related picocyanobacteria groups."

Paragraph from L 77: Nested PCR methods are an alternative to single cell genomic approaches. This paragraph could go further in explaining the comparative advantages and disadvantages of the two approaches rather than simply leaving it at cost savings.

We have expanded on the advantages and disadvantages of metabarcoding compared to whole genome sequencing in the same paragraph.

Original: "Metabarcoding is a suitable technique to assess the taxonomic diversity of flow cytometry sorted populations, including *Synechococcus*, because of its high sensitivity (e.g. trace concentrations of DNA can be PCR amplified and sequenced) and lower cost when compared with metagenome or whole genome sequencing."

Edited (Lines 82-91): "Metabarcoding is a suitable technique to assess the taxonomic diversity of flow

cytometry sorted populations with high sensitivity (e.g. trace concentrations of DNA can be PCR amplified and sequenced) and of many samples, including *Synechococcus*. Although alternative approaches of metagenome or whole genome sequencing could similarly obtain the taxonomic diversity without metabarcoding limitations of primer and amplification biases, these approaches require a much larger sequencing depth and sample processing time which would increase the cost and time needed (19). Whole genome sequencing would instead be more advantageous when applied to functional diversity and population genetics (20, 21)."

Paragraph from L 102: This paragraph could be improved by outlining some of the risks and disadvantages of nested PCR (i.e., errors induced from double amplification that would manifest as overestimations of diversity at the ASV-level) in the context of how these risks are mitigated and why the pros outweigh the cons. Alternatively, this material could be more clearly articulated in the Discussion.

We outlined the risks of nested PCR compared to standard PCR in the paragraph from line 321 in the discussion. Based on existing literature directly comparing nested PCR to standard PCR, there is no consensus on whether nested PCR would result in over or under-estimations of diversity, as previous two studies reported lower diversity in nested PCR approaches while this study obtained a higher diversity. To reduce the risks of misclassifying chimeras as ASVs and therefore over representing diversity, we compared the translated protein sequences of ASVs against the reference database. Based on this analysis, we concluded that our nested PCR improved the specificity compared to standard PCR instead, likely due to two separate sets of oligonucleotide primers to the template DNA.

L162: these "oceanographic patterns" should be cited to establish this line of reasoning

The term "oceanographic patterns" in this sentence is used to highlight the observed pattern of community composition in this study across subtropical to subantarctic waters, and not the physical and chemical oceanographic patterns. We have changed the term to clarify this sentence.

Original: "Yet, oceanographic patterns such as the higher relative abundance of clade IVa in ST compared to SA waters was captured by both approaches."

Edited (Lines 172–174): "Yet, patterns of community composition such as the higher relative abundance of clade IVa in ST compared to SA waters were captured by both approaches."

L243-244: This conclusion is not justified because spurious amplification is not the only reason that an ASV will not match a given database. Please put this statement into the context and limitations of databases in general.

We agree with the reviewer and have corrected the sentence to include the limitations of databases.

Original: "More than half of these ASVs had no sequence match in the database, indicating that they were likely to have resulted from spurious amplification."

Edited (Lines 254-257): "More than half of these ASVs had no sequence match in the database, indicating that they could have resulted from spurious amplification or the current database did not have a corresponding reference sequence."

Minor comment: There are several mistakes in the language and grammar that can easily be fixed via revisions. Therefore, I will not go note every instance.

We have corrected the mistakes in language and grammar.

References

1. **Heath RA**. 1983. Observations on Chatham Rise Currents. New Zealand J Mar Freshw Res 17 (3):321–330. doi: 10.1080/00288330.1983.9516006.
2. **Chiswell SM**. 1994. Acoustic Doppler Current Profiler Measurements over the Chatham Rise. New Zealand J Mar Freshw Res 28 (2):167–178. doi:10.1080/00288330.1994.9516605.
3. **Stramma L, Peterson RG, Tomczak M**. 1995. The South Pacific Current. J Phys Oceanogr 25 (1):77–91. doi: 10.1175/1520-0485(1995)025¡0077:TSPC¿2.0.CO;2.
4. **Lüskow F, Pakhomov EA, Stukel MR, Décima M**. 2020. Biology of Salpa Thompsoni at the Chatham Rise, New Zealand: Demography, Growth, and Diel Vertical Migration. Mar Biol 167 (12):175. doi:10.1007/s00227-020-03775-x.
5. **Boyd P, LaRoche J, Gall M, Frew R, McKay RML**. 1999. Role of Iron, Light, and Silicate in Controlling Algal Biomass in Subantarctic Waters SE of New Zealand. J Geophys Res Ocean 104 (C6):13395–13408. doi: 10.1029/1999JC900009.
6. **Hall JA, James MR, Bradford-Grieve JM**. 1999. Structure and Dynamics of the Pelagic Microbial Food Web of the Subtropical Convergence Region East of New Zealand. Aquat Microb Ecol 20 (1):95–105. doi:10.3354/ame020095.
7. **Lomas MW, Baer SE, Mouginot C, Terpis KX, Lomas DA, Altabet MA, Martiny AC**. 2021. Varying Influence of Phytoplankton Biodiversity and Stoichiometric Plasticity on Bulk Particulate Stoichiometry across Ocean Basins. Commun Earth & Environ 2 (1):1–10. doi:10.1038/s43247-021-00212-9.
8. **Hansman RL, Sessions AL**. 2016. Measuring the in Situ Carbon Isotopic Composition of Distinct Marine Plankton Populations Sorted by Flow Cytometry. Limnol Oceanogr Methods 14 (2):87–99. doi:10.1002/lom3.10073.
9. **Casey JR, Lomas MW, Michelou VK, Dyhrman ST, Orchard ED, Ammerman JW, Sylvan JB**. 2009. Phytoplankton Taxon-Specific Orthophosphate (Pi) and ATP Utilization in the Western Subtropical North Atlantic. Aquat Microb Ecol 58 (1):31–44. doi:10.3354/ame01348.
10. **Duhamel S, Van Wambeke F, Lefevre D, Benavides M, Bonnet S**. 2018. Mixotrophic Metabolism by Natural Communities of Unicellular Cyanobacteria in the Western Tropical South Pacific Ocean. Environ Microbiol 20 (8):2743–2756. doi:10.1111/1462-2920.14111.
11. **Berthelot H, Duhamel S, L'Helguen S, Maguer JF, Wang S, Cetinić I, Cassar N**. 2019. NanoSIMS Single Cell Analyses Reveal the Contrasting Nitrogen Sources for Small Phytoplankton. The ISME J 13 (3):651–662. doi: 10.1038/s41396-018-0285-8.
12. **Aldunate M, Henríquez-Castillo C, Ji Q, Lueders-Dumont J, Mulholland MR, Ward BB, von Dassow P, Ulloa O**. 2020. Nitrogen Assimilation in Picocyanobacteria Inhabiting the Oxygen-Deficient Waters of the Eastern Tropical North and South Pacific. Limnol Oceanogr 65 (2):437–453. doi:10.1002/lno.11315.
13. **Li WKW**. 1994. Primary Production of Prochlorophytes, Cyanobacteria, and Eucaryotic Ultraphytoplankton: Measurements from Flow Cytometric Sorting. Limnol Oceanogr 39 (1):169–175. doi:10.4319/lo.1994.39.1.0169.
14. **Rii YM, Duhamel S, Bidigare RR, Karl DM, Repeta DJ, Church MJ**. 2016. Diversity and Productivity of Photosynthetic Picoeukaryotes in Biogeochemically Distinct Regions of the South East Pacific Ocean. Limnol Oceanogr 61 (3):806–824. doi:10/f8nf3k.
15. **Jardillier L, Zubkov MV, Pearman J, Scanlan DJ**. 2010. Significant CO2 Fixation by Small Prymnesiophytes in the Subtropical and Tropical Northeast Atlantic Ocean. The ISME J 4 (9):1180–1192. doi:10.1038/ismej.2010.36.
16. **Hartmann M, Gomez-Pereira P, Grob C, Ostrowski M, Scanlan DJ, Zubkov MV**. 2014. Efficient CO2 Fixation by Surface *Prochlorococcus* in the Atlantic Ocean. The ISME J 8 (11):2280–2289. doi:10.1038/ismej.2014.56.
17. **Farrant GK, Doré H, Cornejo-Castillo FM, Partensky F, Ratin M, Ostrowski M, Pitt FD, Wincker P, Scanlan DJ, Iudicone D, Acinas SG, Garczarek L**. 2016. Delineating Ecologically Significant Taxonomic Units from Global Patterns of Marine Picocyanobacteria. Proc Natl Acad Sci 113 (24):E3365–E3374. doi:10.1073/pnas.1524865113.
18. **Doré H, Leconte J, Guyet U, Breton S, Farrant GK, Demory D, Ratin M, Hoebeke M, Corre E, Pitt FD, Ostrowski M, Scanlan DJ, Partensky F, Six C, Garczarek L**. 2022. Global Phylogeography of Marine *Synechococcus* in Coastal Areas Reveals Strikingly Different Communities than in the Open Ocean. bioRxiv.
19. **Santoferrara L, Burki F, Filker S, Logares R, Dunthorn M, McManus GB**. 2020. Perspectives from Ten Years of Protist Studies by High-Throughput Metabarcoding. J Eukaryot Microbiol 67 (5):612–622. doi:10.1111/jeu.12813.

20. **Malmstrom RR, Rodrigue S, Huang KH, Kelly L, Kern SE, Thompson A, Roggensack S, Berube PM, Henn MR, Chisholm SW**. 2013. Ecology of Uncultured *Prochlorococcus* Clades Revealed through Single-Cell Genomics and Biogeographic Analysis. The ISME J 7 (1):184–198. doi:10.1038/ismej.2012.89.
21. **Santoro AE, Kellom M, Laperriere SM**. 2019. Contributions of Single-Cell Genomics to Our Understanding of Planktonic Marine Archaea. Philos Trans Royal Soc B: Biol Sci 374 (1786):20190096. doi:10.1098/rstb.2019.0096.

January 30, 2023

Mx. Denise Rui Ying Ong
Nanyang Technological University
50 Nanyang Avenue
SIngapore
Singapore

Re: Spectrum04086-22R1 (Nested PCR approach for *petB* gene metabarcoding of marine *Synechococcus*)

Dear Mx. Denise Rui Ying Ong:

Your manuscript has been accepted, and I am forwarding it to the ASM Journals Department for publication. You will be notified when your proofs are ready to be viewed.

The ASM Journals program strives for constant improvement in our submission and publication process. Please tell us how we can improve your experience by taking this quick Author Survey.

Publication Fees: We have partnered with Copyright Clearance Center to collect author charges. You will soon receive a message from no-reply@copyright.com with further instructions. For questions related to paying charges through RightsLink, please contact Copyright Clearance Center by email at ASM_Support@copyright.com or toll free at +1.877.622.5543. Hours of operation: 24 hours per day, 7 days per week. Copyright Clearance Center makes every attempt to respond to all emails within 24 hours. For a complete list of **Publication Fees**, including supplemental material costs, please visit ourwebsite.

ASM policy requires that data be available to the public upon online posting of the article, so please verify all links to sequence records, if present, and make sure that each number retrieves the full record of the data. If a new accession number is not linked or a link is broken, provide production staff with the correct URL for the record. If the accession numbers for new data are not publicly accessible before the expected online posting of the article, publication of your article may be delayed; please contact the ASM production staff immediately with the expected release date.

Corresponding authors may join or renew ASM membership to obtain discounts on publication fees. Need to upgrade your membership level? Please contact Customer Service at Service@asmusa.org.

Thank you for submitting your paper to Spectrum.

Sincerely,

Blaire Steven
Editor, Microbiology Spectrum

Journals Department
American Society for Microbiology
1752 N St., NW
Washington, DC 20036
E-mail: spectrum@asmusa.org
